# Clever Cats: Do They Utilize Change Blindness as a Covered Approaching Strategy?

**DOI:** 10.1177/2041669521994597

**Published:** 2021-02-20

**Authors:** Alexander Pastukhov, Claus-Christian Carbon

**Affiliations:** Department of General Psychology and Methodology, 14310 University of Bamberg, Bamberg, Germany; Forschungsgruppe EPÆG (Ergonomics, Psychological Æsthetics, Gestalt), Bamberg, Bavaria, Germany

**Keywords:** attention, change blindness, inattention/attention blindness, tracking/shifting attention

## Abstract

Sometimes, we do not notice big changes in our environment, if these changes occur while we perform eye movements or external events interrupt our perception. This striking phenomenon is known as “change blindness.” Research on chimpanzees, macaques, and pigeons suggests that change blindness may not be unique to humans, but our understanding is limited by the difficulty of carrying out change blindness experiments in animals. However, let’s have a look to the habitats of some of our most beloved four-legged friends: cats and dogs. Here, we list several online videos with cats and a husky appear to use humans’ change blindness to their advantage to sneak upon them. Thus, we might be able to deduce the effects of change blindness and other perceptual phenomena from animals’ behaviour. Our clear message: Watch more (cat) videos! Watch them as perceptual scientists by means of observing and analysing the cat’s behaviour.

Change blindness refers to our occasional inability to notice surprisingly large changes in the world (Jensen et al., 2011). Most of us were acquainted with the phenomenon in our childhood when looking to find five differences (Figure 1). What makes the task challenging are the saccades that interrupt your perception and prevent you from easily detecting the differences (Grimes, 1996). A better known and far more widely used approach is a flicker paradigm when two photographs are presented intermittently with a brief blank screen in-between (Rensink et al., 1997). What makes this phenomenon so counterintuitive is the sheer salience of the difference once it has been spotted. The discovered change is so obvious that it is hard to understand, why it was so difficult to find it just seconds ago. More importantly, it reflects the limitation of our perceptual and cognitive system rather than the artificial nature of the flicker task (Carbon, 2014). The same blindness to large changes in the environment occurs in naturalistic settings, for example, while talking to a stranger (Simons & Levin, 1998), inspecting the stranger’s face (Utz & Carbon, 2020), or even making tea (Tatler, 2001). This implies that we experience change blindness daily (and, most probably, many times a day) but fail to notice it, as there is no experimenter to inform us about it and no solution is provided on the back pages.

**Figure 1.**
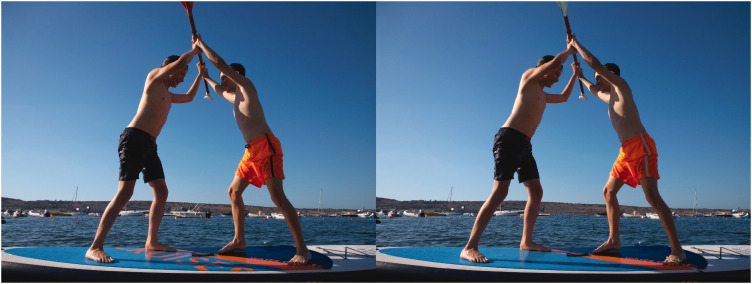
Spot five differences between two pictures.

Given how profound effects of change blindness are in humans, it is natural to ask whether animals also experience it. Indeed, similar conditions do impair their performance (Herbranson, 2019). For example, chimpanzees exhibited similar difficulties in a flicker-type task (Tomonaga & Imura, 2015). Similarly, macaques have difficulty detecting changes both in one-shot change detection (Heyselaar et al., 2011) and a flicker paradigm task (Chau et al., 2011). The phenomenon is not specific to mammals, as pigeons also exhibit change blindness (Herbranson & Davis, 2016). Although there might be some exceptions (Leinwand & Brosnan, 2019), change blindness appears to reflect some common limitation shared across branches of the evolutionary tree.

It would be informative to have a more detailed catalogue of species that exhibit change blindness, but the progress is slowed down by laborious and time-consuming training. However, there might be an appealing alternative to that: Observing animals in their habitats. Later, we list several example videos where cats (and a husky) appear to rely on change blindness to sneak up on their owners. In these videos, animals “freeze” every time the camera and the owner’s gaze are directed at them. The resultant motion creates a cat-made change blindness flicker task. You are unlikely to experience change blindness when watching these videos for the sole reason that cats are leading actors and the camera and, therefore, your attention is directed at them. However, the same sneak-crouch tactics is used by larger cats when stalking their prey (Elliott et al., 1977). In that case, spotting a sneaking predator becomes a true change blindness task that the typical prey—zebras and gazelles—may not solve before it is too late. Thus, it could tell us which animals share change blindness with humans without a need for direct experiment.

In short, the videos show that change blindness can be studied indirectly by examining the hunting strategies of various animals. They also remind us that even descriptive knowledge of animal behaviour can advance our understanding of common traits in perception. Also, keep in mind that the internet became a rich source of documented observations and of actual behavioural experiments, such as the “fluff challenge” that tested the owner’s permanence in pets (https://youtu.be/ubgmp80r8PM). It is up to us, as perceptual scientists, to decipher these phenomena and learn to understand and explain the underlying mechanisms.

*Example videos of sneaky cats*

- https://youtu.be/MEmEN8PFsgI
- https://youtu.be/ON37cSSTj8A
- https://youtu.be/hWN1jCwXLYk
- https://youtu.be/EzK2AAR7_1k
- https://gfycat.com/reasonabledensearieltoucan-sneaky-cat

*And not to forget: example video of a smartly approaching husky*

- https://youtu.be/PjHCXn_T2BQ
